# Increased endothelial biomarkers are associated with HIV antiretroviral therapy and C-reactive protein among a African rural population in Limpopo Province, South Africa

**DOI:** 10.3389/fpubh.2022.980754

**Published:** 2022-11-04

**Authors:** Sidney Hanser, Peter Modupi Mphekgwana, Mpho Matthias Moraba, Lourens Erasmus, Marlise van Staden

**Affiliations:** ^1^Department of Physiology and Environmental Health, University of Limpopo, Polokwane, South Africa; ^2^Research Administration and Development, University of Limpopo, Polokwane, South Africa; ^3^Department of Medical Science, University of Limpopo, Polokwane, South Africa

**Keywords:** endothelial activation, endothelial dysfunction, sICAM-1, sVCAM-1, L-Selectin, CRP, HIV, HAART

## Abstract

In Sub-Saharan Africa (SSA) endothelial dysfunction (ED) and chronic inflammation in the HIV-positive adults population who are on highly active antiretroviral therapy (HAART) are not fully explored. We determined the effect of HAART on chronic inflammation and ED among HAART-exposed adults in a rural setting. Weight and height were measured to quantify the body mass index (BMI). Lipid and Glucose levels were determined. C-reactive protein (CRP), L-selectin, soluble intercellular adhesion molecule (sICAM-1), and soluble vascular cell adhesion molecule (sVCAM-1) in serum samples were tested. The majority of the HAART-exposed group were on treatment for <5 years. Soluble intercellular adhesion molecules, sVCAM-1, L-selectin and CRP were elevated in the HIV-infected groups as compared to the control group. The multivariate analysis showed that HIV infection (HAART-naïve) associated with increased sICAM-1 (β = 0.350; 95% CI: 0.035–0.664, *p* = 0.029) and L-selectin (β = 0.236; 95% CI: 0.038–0.434, *p* = 0.019) but not sVCAM-1 (β = 0.009; 95% CI: 0.252–0.270, *p* = 0.468). The HAART-exposed group is associated with sVCAM-1 (β = 0.250; 95% CI: 0.015–0.486, *p* = 0.037) but not with sICAM-1- (β = 0.253; 95% CI: −0.083–0.590, *p* = 0.14) and L-selectin (β = 0.119; 95% CI: −0.016–0.253, *p* = 0.084). sVCAM-1 was associated with decreased alcohol consumption (β = −0.245; 95% CI: −0.469–0.021, *p* = 0.032) while L-selectin was associated with decreased total cholesterol (β = −0.061; 95% CI: −0.124–0.002, *p* = 0.05) and increased CRP (β = 0.015; 95% CI: 0.009–0.022, *p* < 0.001). Increased endothelial biomarkers were associated with HIV disease and HAART in a rural black adult population of African descent after controlling for CVD risk factors. Inflammation (as measured with CRP) may play an important role in endothelial activation. Further studies are needed to explore the association between endothelial dysfunction and inflammation especially among the HIV-positive population on HAART in similar settings.

## Introduction

Cardiovascular disease (CVD) risk is increased among human immunodeficiency (HIV) infection and it persists even during highly active antiretroviral therapy (HAART) mediated viral suppression (1, 2). Endothelial dysfunction is well-known in several CVD and is a early event in the development atherosclerosis (3, 4). Evidence exists that elevated levels of endothelial biomarkers [soluble intercellular cell adhesion molecule (sICAM-1), soluble vascular cell adhesion molecule (sVCAM-1), L-selectin] are indicative of inflammation, endothelial dysfunction (ED) and may serve as predictors of atherosclerosis and various cardiovascular complications (5, 6). The endothelium plays a crucial role in the maintainance of vascular and metabolic homeostasis (4, 7, 8).

The endothelium plays a critical role in enhancing normal blood perfusion by creating an anti-inflammatory and non-adhesive surface area (9–11). Thus, any perturbations in the vascular endothelium homeostasis as a result of diverse stimuli, such as HIV infection or adverse effects of HAART may result in ED. Endothelial dysfunction is present in several CVD risk factors which involve metabolic disorders such as hypercholesterolemia, insulin resistance (IR), type 2 diabetes mellitus (T2DM), and lifestyle risk factors such as alcohol and tobacco consumption (12–17). Endothelial dysfunction also correlates with unfavorable outcomes in patients with CVD such as myocardial infarction (MI) (5, 18). Endothelial activation, ED and inflammation plays a critical role in the development of CVD and several VCAM-1, ICAM-1, and L-selectin have been reported as biomarkers of ED in atherosclerosis (5, 6, 17, 19–21).

Studies have showed that chronic HIV inflammation may cause endothelial activation and ED which have been proposed as a potential mechanism for HIV and HAART-induced atherosclerosis (22, 23). Inflammation may lead to the release of pro-inflammatory cytokines, such as interleukin-6 (IL-6), CRP, and tumor necrosis factor alpha (TNF-?) which can cause increased expression of selectins and cell adhesion molecules (CAMs) on the surface of the endothelium (24). Endothelial activation has been previously confirmed among HIV-positive populations in Sub-Saharan Africa (SSA) and this persisted even after the initiation of HAART (23, 24). Few studies in the SSA region confirmed HIV-associated endothelial activation and dysfunction while the majority of studies are observed in non-African populations (25–27). This, therefore, necessitated studies exploring the association between endothelial function, HIV disease and antiretroviral (ARV) therapy in the SSA. Since ED is a precursor event of atherosclerotic disease, it is considered a powerful diagnostic tool to predict CVD (5, 6, 28, 29).

In this rural black South African population, the aim of this study was to: (1) determine ED in HIV-positive individuals (HAART or HAART-naïve) and HIV-negative participants; (2) evaluate whether endothelial biomarkers (sICAM-1, sVCAM-1, and L-selectin) are independently associated with HIV status; (3) to assess whether inflammatory biomarker (CRP) is associated with endothelial biomarkers.

## Materials and methods

### Study design and procedures

The cross-sectional study was carried out from January 2017 and March 2019 in the Mankweng hospital under Polokwane Municipality located within the Capricorn District of Limpopo Province. Ethical clearance was obtained from the University of Limpopo Turfloop Research and Ethics Committee (TREC/119/2016:PG) and permission was further granted by the Department of Health and Primary Health Care and Social Development to conduct this study. The study protocol complied with the Declaration of Helsinki as revised in 2013.

We conveniently and purposively selected the Mankweng daycare clinic which falls within Mankweng. The clinic offers services such as family planning, disease detection, immunization, treatment, and management of various diseases. Permission was obtained from the Department of Health (Capricorn District) to conduct this study. The Limpopo Province HIV prevalence was estimated at 8.3% in 2015/2016 among men and women between 15 and 49 years (30). The sample size (*n* = 113) was calculated using the mathematical formula developed by Cochran (31) with a 5% error and a 95% confidence level. A total of 158 participants (100 females and 58 males) who visited the Mankweng day-care clinic between January 2017 and March 2019 were recruited. The total study participants (*n* = 158) consisted of HIV positive participants who were exposed HAART (*n* = 71), HAART-naïve (*n* = 36) and HIV negative (*n* = 51). Consent were requested from all participants and and voluntary HIV testing and counseling was provided by qualified health care practitioners. Individuals with cardiometabolic disorders such as T2DM, hypertension, or dyslipidaemia participants taking medication for cardiovascular-related conditions, women who are pregnant, breastfeeding and co-infections such as tuberculosis (TB) were excluded from the study.

A structured questionnaire was used to gather demographic information (age, gender and ethnicity), HIV status, medical history, type of regimen, alcohol and tobacco consumption. The questionnaire was also designed to record blood pressure and all the anthropometric measurements, and it was available in English and Sepedi (common language in Mankweng District). The systolic blood and diastolic blood pressure were determined using the digital automated Omron M2 blood pressure monitors (Omron Healthcare, Japan). The weight was measured using the electronic body weight scale (Pee Pee Electricals; Delhi, India) and height was measured using the stadiometer (Seca GmbH, & Co. KG, Germany) according to the International Standards for Anthropometric Assessment of the International Society for Advancement of Kinanthropometry (ISAK) (32). The waist circumference (WC) was quantified with the circumference tape measurer (Seca GmbH, & Co. KG, Germany) following the WHO measurement protocol (33).

Fasting venous blood samples were acquired from all the participants. Serum and plasma samples were centrifuged for 20 min at 3,000 revolutions per minute (RPM) according to the appropriate methods. The whole blood samples were immediately used and the serum and plasma were stored at −80°C in the laboratory until further analysis. Participants received counseling before and after HIV testing by a registered counselor. The HIV status of all participants were determined by the serum sample which were performed at the Medical Sciences Laboratory (University of Limpopo) (Alere Determine HIV-1/2, Alere to Abbott Medical Co Ltd., Japan). The CD4+ count in the whole blood samples was determined with the use of Cytomics FC500 Flow Cytometer Multi-Platform loader (MPL) which is an automated tube-based acquisition device for clinical assays at the Lancet Laboratories in Polokwane (Beckman Coulter FC500 MPL/CellMek, Miami, FL). Quantitative determination of glucose, high-density lipoprotein cholesterol (HDL-C), triglycerides (TG) and glucose concentrations in the serum of the participants was done with the Cobas^®^ Integra 400 plus auto-analyser (Roche Holding AG, Basel, Switzerland). The Laboratory information system was used to quantify the low density lipoprotein cholesterol (LDL-C levels.

Bead-based multiplex kits were acquired to quantify the 4 serum biomarkers on a Luminex 200^TM^ device. The human CVD magnetic bead panel 2 were used to determine C-reactive protein and L-selectin concentrations simultaneously in serum samples (EMD Millipore Corporation, Billerica, USA, 2017). The human CVD magnetic bead panel 3 were acquired to determine sICAM-1 and sVCAM-1 concentrations in serum samples (Merck Millipore Corporation, Billerica, MA, USA, 2017). Luminex 200^TM^ instrument system with xPonent 4.2 software (Merck KGaA, Germany) was used to analyse all the biomarkers (bead-based multiplex kits) of interest. All the biomarkers were analyzed at the Center for Vaccines and Immunology under National Institute for Disease Control (NICD) in South Africa.

### Statistical analysis

All variables underwent descriptive statistical analysis. Variables were presented as mean ± standard deviation (SD) and categorical variables were expressed as frequency and percentages. The *t-*test and one-way analysis of variance (ANOVA) was used to compare the significant differences between the groups. The Shapiro-Wilk test and Gaussian distribution curves were used to test for normality. All the endothelial biomarkers (sVCAM, sICAM and L-section) were not normally distributed. After taking the log transformation, the endothelial biomarkers were now normally distributed. Multiple linear regression analysis was applied to determine the relationship between CVD risk factors (HIV status, smoking, alcohol consumption, Cholesterol, CRP and mean arterial pressure) and endothelial biomarkers (sVCAM, sICAM and L-section). The significance difference and association level were assumed at *p* < 0.05. All the data were analyzed using the IBM statistical package of the social sciences (SPSS) (IBM, Chicago, USA) version 27.

## Results

### General characteristics of the study participants

The study participants (*n* = 158), with a mean age (±SD) of 40.23 ± 12.72 years, consist mainly of an African Black ethnic group (100%) with more females (63.3%) as compared to males (36.7%) (Table 1). The general characteristics of the study participants included three groups respectively, where the control group consisted out of 51 (31.9%), the HAART-naïve group consisted out of 36 (22.5%) and the HAART-exposed group consisted out of 73 (45.6%) (Table 1). The majority of the HAART-exposed participants were on an NNRTI-based regimen (80.0%). Moreover, 60.3% of the patients enrolled were on HAART treatment for <5 years (Table 2). The HAART-exposed group showed significantly higher HDL-C levels compared to both the HAART-naïve and control groups (Table 3). The LDL-C levels were significantly higher in the control group compared to the HAART-naïve group (Table 3). Unadjusted analysis for the endothelial biomarkers, L-selectin (*p* = 0.010) and sICAM-1 (*p* = 0.017) was significantly higher in the HAART-naïve group compared to HAART-exposed and control group (Table 4). However, no significant difference was observed for sVCAM-1 (*p* = 0.123) across the three groups (Table 4). CRP was also significantly higher in the HAART-naïve group (*p* = 0.007) as compared to HAART-exposed and control group (Table 4).

**Table 1:** Demographics of the study participants.

	**Total group**	**HAART-exposed**	**HAART-naïve**	**HIV negative**	***p*-Value**
	***n* = 158**	***n* = 71**	***n* = 36**	***n* = 51**	
**Ethnicity (Black) (** ***n*** **, %)**	158 (100)	–	–	–	–
**Age, years**	40.23 ± 12.72	43.24 ± 10.19	39.06 ± 11.70	36.88 ± 15.53	**0.019**
**Gender**					
Female, *n* (%)	100 (63.3)	46 (64.8)	20 (55.6)	34 (66.7)	0.536
Male, *n* (%)	58 (36.7)	25 (35.2)	16 (44.4)	17 (33.3)	

HAART, highly active antiretroviral therapy.

Data expressed as mean ± standard deviation or sum and percentage.

One-way analysis of variance, Bold text indicates *p* < 0.05.

**Table 2:** HIV and HAART-related characteristics of the study participants.

	**Total Group**	**HAART-exposed**	**HAART-naïve**	**HIV negative**	***p*-Value**
	***n* = 158**	***n* = 71**	***n* = 36**	***n* = 51**	
**HIV related parameters**					
NNRTIs use (*n*, %)	–	57 (80.0)	–	–	–
PIs use (*n*, %)	–	14 (20.0)	–		–
CD4^+^ cell count (cells/μl)	–	433.02 (± 214.04)	341 (± 281.44)	–	**0.053**
**HAART duration (** ***n*** **, %)**					
<1 year	–	16 (23.5%)	–	–	–
1–2.9 years	–	13 (19.2%)	–	–	–
3–4.9 years	–	12 (17.6%)	–	–	–
≥ 5 years	–	27 (39.7%)	–	–	–

NNRTIs, non-nucleotide reverse transcriptase inhibitors; PIs, protease inhibitors; HAART, highly active antiretroviral therapy.

Data presented as mean ± standard deviation or sum and percentage.

**Table 3:** CVD risk factors of the study participants.

	**Total Group**	**HAART-exposed**	**HAART-naïve**	**HIV negative**	***p*-Value**
	***n* = 158**	***n* = 71**	***n* = 36**	***n* = 51**	
**Body mass index (kg/m** ^**2**^ **)**	25.75 ± 5.82	25.71 ± 5.97	24.10 ± 5.21	27.00 ± 5.82	0.071
**Cardiovascular measurements (mm Hg)**					
SBP	121.0 ± 17.86	123.69 ± 21.42	119.67 ± 14.67	118.19 ± 13.65	0.216
DBP	75.02 ± 9.76	76.23 ± 10.59	74.19 ± 8.97	73.92 ± 9.06	0.373
MAP	90.35 ± 11.60	92.05 ± 13.46	89.35 ± 10.10	88.68 ± 9.49	0.243
**Lifestyle risk factors (** ***n*** **, %)**					
Alcohol users	33 (21)	18 (25.3)	10 (27.8)	5 (10)	0.058
Tobacco users	36 (22.7)	18 (25.3)	11 (30.6)	7 (19.4)	0.407
**Biochemical measurements**					
Glucose (mmol/L)	5.21 ± 1.56	5.44 ± 1.95	5.09 ± 1.45	4.95 ± 0.91	0.214
HDL-C (mmol/L)	1.35 ± 0.42	1.45 ± 0.35	1.22 ± 0.50	1.31 ± 0.44	**0.024**
LDL-C (mmol/L)	2.36 ± 0.92	2.47 ± 0.87	1.87 ± 0.80	2.55 ± 0.98	**0.001**
TC (mmol/L)	4.28 ± 1.05	4.49 ± 0.98	3.66 ± 0.87	4.43 ± 1.13	**0.001**

HAART, highly active antiretroviral therapy; SBP, systolic blood pressure; DBP, diastolic blood pressure; MAP, mean arterial pressure; HDL-C, high-density lipoprotein cholesterol; LDL-C, low-density lipoprotein cholesterol, TC, Total Cholesterol.

Data presented as arithmetic mean ± standard deviation, count and percentage.

Bold text indicates *p* < 0.05. A Self-reported smoking and alcohol use. One-way analysis of variance (ANOVA).

**Table 4:** Biomarkers of the study participants.

	**Total group**	**HAART-exposed**	**HAART-naïve**	**HIV negative**	***p*-Value**
	***n* = 158**	***n* = 71**	***n* = 36**	***n* = 51**	
C-reactive protein (ng/mL	0.71 (2.05–0.20)	0.87 (3.90–0.27)	0.90 (2.71–0.26)	0.36 (1.05–0.06)	**0.007**
L-selectin (ng/mL)	0.06 (0.11–0.03)	0.06 (0.13–0.04)	0.09 (0.17–0.05)	0.05 (0.08–0.03)	**0.010**
sICAM-1 (ng/mL)	0.71 (1.4–0.28)	0.68 (2.11–0.26)	1.00 (1.56–0.65)	0.49 (0.98–0.19)	**0.017**
sVCAM-1 (ng/mL)	6.83 (11.754.16)	7.46 (11.12–5.19)	6.15 (9.65–0.92)	6.68 (13.47–98)	0.123

HAART, highly active antiretroviral therapy; sICAM-1, soluble intracellular molecule; sVCAM-1, soluble vascular cell adhesion molecule.

Data presented as median and interquartile range. One-way analysis of variance (ANOVA), Bold text indicates *p* < 0.05.

In adjusted analysis, after controlling for CVD risk factors. HIV-naïve group was significantly associated with increased log sICAM (β = 0.350; 95% CI: 0.035–0.664, *p* = 0.029) and increased log L-selectin (β = 0.236; 95% CI: 0.038–0.434, *p* < 0.001) but not with log sVCAM-1 (β = 0.009; 95% CI: 0.252–0.270, *p* = 0.468) (Table 5). Further, HAART-exposed group was significantly associated with increased log sVCAM (β = 0.250; 95% CI: 0.015–0.486, *p* = 0.037) but not with log sICAM (β = 0.253; 95% CI: −0.083–0.590, *p* = 0.14) and log L-selectin (β = 0.119; 95% CI: −0.016–0.253, *p* = 0.084) (Table 5). Alcohol consumption was only associated with decreased log sVCAM (β = −0.245; 95% CI: −0.469– −0.021, *p* = 0.032), however, no association was observed with log sICAM (β = −0.107, 95% CI: −0.435–0.221), *p* = 0.524) and log L-selectin (β = 0.090, 95% CI: −0.080–0.260, *p* = 0.301). Cholesterol significantly associated with decreased log L-selectin (β = −0.061; 95% CI = −0.124–0.002, *p* = 0.05) but not with log sVCAM-1 (β = 0.082, 95% CI: −0.011–0.175, *p* = 0.083) and log sICAM-1 (β = 0.081, 95% CI: −0.069–0.230, *p* = 0.291). CRP associated with increased log L-selectin (β = 0.015; 95% CI: 0.009–0.022, *p* < 0.001) but not with sVCAM-1 (β = 0.004; 95% CI: 0.009–0.001, *p* = 0.001) and sICAM-1 (β = 0.004; 95% CI: −0.010–0.003, *p* = 0.305).

**Table 5:** Adjusted multivariate analysis between endothelial biomarkers (sVCAM, sICAM and L-section) and CVD risk factors for the entire study participants).

**Variables**	**Log sVCAM** **Coefficient (95% CI)**	***P*-value**	**Log SICAM** **Coefficient (95% CI)**	***P*-value**	**Log L-section** **Coefficient (95% CI)**	***P*-value**
HIV Status (ref = Negative)						
HIV-naïve	0.009 (−0.252; 0.270)	0.468	0.350 (0.035; 0.664)	**0.029**	0.236 (0.038; 0.434)	**0.019**
HIV-treatment	0.250 (0.015; 0.486)	**0.037**	0.253 (−0.083; 0.590)	0.140	0.119 (−0.016; 0.253)	0.084
Gender (ref = Female)	0.004 (−0.215; 0.224)	0.968	0.126 (−0.177; 0.430)	0.415	0.052 (−0.091; 0.195)	0.477
Age	−0.003 (−0.012; 0.005)	0.468	0.001 (−0.008; 0.010)	0.831	0.001 (−0.005; 0.004)	0.854
Smoke (ref = No)	−0.177 (−0.408; 0.054)	0.132	−0.081 (−0.426; 0.264)	0.646	−0.015 (−0.179; 0.149)	0.856
Alcohol consumption (ref = No)	−0.245 (−0.469; −0.021)	**0.032**	−0.107 (−0.435; 0.221)	0.524	0.090 (−0.080; 0.260)	0.301
Total cholesterol	0.082 (−0.011; 0.175)	0.083	0.081 (−0.069; 0.230)	0.291	−0.061 (−0.124; 0.002)	**0.050**
C-reactive protein	−0.004 (−0.009; 0.001)	0.094	−0.004 (−0.010; 0.003)	0.305	0.015 (0.009; 0.022)	**< 0.001**
MAP	−0.005 (−0.014; 0.004)	0.256	0.001 (−0.010; 0.013)	0.832	0.002 (−0.004; 0.007)	0.590

sICAM-1, soluble intracellular molecule; sVCAM-1, soluble vascular cell adhesion molecule; MAP, Mean Arterial Pressure.

Bold text indicates *p* < 0.05. CI, Confidence Intervals.

## Discussion

In the present study, HIV status was associated with increased biomarkers of endothelial dysfunction compared to the HIV-negative control, after controlling for CVD risk factors. This finding, therefore, complements the limited reports in Sub-Saharan Africa (23) and is consistent with findings from high-income countries (34). HIV-infected participants, who had never received ART, independently associated with increased serum levels of sICAM-1. Our study did not observe any association between HIV naive and sVCAM-1 as reported previously which may be attributed to locality and gender disparities (35, 36). However, the latter studies (35, 36) support our findings on elevated levels of sICAM-1 among black South Africans and Kenyan women, respectively, following HIV infection. Our findings are further also consistent with high-income countries (37). The mechanism for increased elevation in the present study may be related to inflammation since it was previously reported that pro-inflammatory cytokines, such as IL-6, CRP, and TNF-? are released during HIV-infection and can cause increased expression of CAMs on the surface of the endothelium (26, 36–38). We further observed that HIV-infected participants were independently associated with increased L-selectin. It has been previously reported that L-selectin mediates monocyte attachment to human activated endothelium (39). CRP is a systemic inflammatory marker where it was previously associated with HIV infection (40). Elevated L-selectin has been associated with inflammation (41). The association between HIV infection and L-selectin in the current study may be attributed to chronic inflammation. Chronic HIV inflammation is known to induce endothelial activation and it has been suggested as a possible mechanism for HIV-induced atherosclerosis (22, 42). Further, in the current study, CRP was positively associated with increased L-selectin, which may reiterate the role of inflammation in endothelial activation.

HAART-exposed participants were also associated with an increased sVCAM-1 after controlling for traditional CVD risk factors. Our findings are in support of previous studies performed in South Africa (23), Kenya (41), Botswana (24), and globally (26) where they also found that endothelial activation persists even after ART initiation. The study's findings further emphasize the strong effect of HIV disease on the pathogenesis of ED even after adjusting for traditional CVD risk factors. sICAM-1 was significantly lower in the HAART-exposed group compared to the HAART-naïve. Further, the lack of association between HIV-treated participants and increased sICAM-1 observed in the current study is contrary to previous findings (24, 36, 41). In addition, a lack of association was also observed between the HAART-exposed participants and L-selectin. These observations in the current study may reflect a beneficial role of HAART on endothelial function. We observed an unexpected negative association between alcohol consumption and sVCAM-1. Further, a negative association was also observed between total cholesterol and L-selectin. We are cautious in interpreting the latter findings of the study. Similar future studies are needed to clarify these findings. More studies are needed to elucidate the mechanisms of ARV drugs on endothelial function which will provide insight into cardiovascular disease in the HIV population. The current cross-sectional study did not permit us to infer causation. The findings of the study were restricted to only black South Africans. Other ethnic groups should also be included in the study to establish any variation in the outcome of this study. Unequal sample groups and a strong gender bias could have influenced the outcome of this study. The gender bias may be due to males being generally the sole provider in the family and hence unable to take time off from work for medical care or to participate in research projects. Also, we were unable to include VL results which could have influenced the outcome of the findings. Future studies should recruit equal amounts of specific ethnic groupings in Limpopo to enhance the quality of conclusions reached. An element of recall bias was evident in this study since there was a level of reliance on information provided by patients and the accuracy of the documented medical files.

## Conclusion

In this rural SSA population in Limpopo province (South Africa), for the first time, we found evidence of ED in the HIV-infected participants despite effective HAART as compared with the HIV-negative participants. HAART associated both positively and negatively with endothelial function after controlling for CVD risk factors. Despite the conflicting findings regarding the association of HIV infection and HAART on the CVD markers, the study succeeded in elucidating that HAART may potentially contribute to endothelial function in this rural black HIV-positive population of African ancestry.

## Data availability statement

The raw data supporting the conclusions of this article will be made available by the authors, without undue reservation.

## Ethics statement

The studies involving human participants were reviewed and approved by University of Limpopo Turfloop Research and Ethics Committee (TREC). The patients/participants provided their written informed consent to participate in this study.

## Author contributions

SH was responsible for the literature search, data analysis, interpretation of data, and writing of the manuscript. SH, MS, MM, LE, and PM were responsible for the conception and design, acquisition and interpretation of data, and for revising the article critically for intellectual content. All authors approved the final version.

## Funding

This research was funded by the Thuthuka Programme of National Research Foundation (Grant No. 107249), Health and Welfare Sector Education and Training Authority (South Africa), and the University of Limpopo (UL).

## Conflict of interest

The authors declare that the research was conducted in the absence of any commercial or financial relationships that could be construed as a potential conflict of interest.

## Publisher's note

All claims expressed in this article are solely those of the authors and do not necessarily represent those of their affiliated organizations, or those of the publisher, the editors and the reviewers. Any product that may be evaluated in this article, or claim that may be made by its manufacturer, is not guaranteed or endorsed by the publisher.
